# Transferrin-targeted magnetic/fluorescence micelles as a specific bi-functional nanoprobe for imaging liver tumor

**DOI:** 10.1186/1556-276X-9-595

**Published:** 2014-10-30

**Authors:** Hui Qi, Zhengzheng Li, Kai Du, Ketao Mu, Qing Zhou, Shuyan Liang, Wenzhen Zhu, Xiangliang Yang, Yanhong Zhu

**Affiliations:** 1 College of Life Science and Technology, Huazhong University of Science and Technology, Wuhan 430074, People's Republic of China; 2 Radiology Department, Tongji Hospital, Huazhong University of Science and Technology, Wuhan 430030, People's Republic of China

**Keywords:** Magnetic resonance imaging, Fluorescence imaging, Target, Micelle, Liver tumor

## Abstract

In order to delineate the location of the tumor both before and during operation, we developed targeted bi-functional polymeric micelles for magnetic resonance (MR) and fluorescence imaging in liver tumors. Hydrophobic superparamagnetic iron oxide nanoparticles (SPIONs) were loaded into the polymeric micelles through self-assembly of an amphiphilic block copolymer poly(ethylene glycol)-poly(ϵ-caprolactone). After, transferrin (Tf) and near-infrared fluorescence molecule Cy5.5 were conjugated onto the surface of the polymeric micelles to obtain the nanosized probe SPIO@PEG-*b*-PCL-Tf/Cy5.5 (SPPTC). Imaging capabilities of this nanoprobe were evaluated both *in vitro* and *in vivo*. The accumulation of SPPTC in HepG2 cells increased over SPIO@PEG-*b*-PCL-Cy5.5 (SPPC) by confocal microscopy. The targeted nanoprobe SPPTC possessed favorable properties on the MR and fluorescence imaging both *in vitro* and *in vivo*. The MTT results showed that the nanoprobes were well tolerated. SPPTC had the potential for pre-operation evaluation and intra-operation navigation of tumors in clinic.

## Background

Magnetic resonance imaging (MRI) has been extensively used in the diagnosis of human cancers for its high spatial resolution [1]. However, it is not sensitive enough to delineate the boundary of tumor clearly. On the other hand, optical imaging, especially near-infrared fluorescence imaging, offers high sensitivity [2,3]. Nevertheless, the low spatial resolution of optical imaging limits its applications in clinical fields [4]. Thus, the combination of MRI and optical imaging can compensate for these drawbacks and provide images of the tumor at both pre-surgical planning stage and surgical resection stage. Such dual modal imaging will fill the gap between pre-operative imaging and intra-operative reality. Superparamagnetic iron oxide nanoparticles (SPIONs) are the well-known MRI contrast agent which can be used to monitor *in vivo* events [5]. Among various fluorescence dyes, quantum dots as excellent fluorescent dyes were limited by their toxicity [6]. Near-infrared (NIR) fluorescence molecule Cy5.5 had been widely utilized in biomedical fields for its high molar absorption coefficient and fluorescence quantum yield, except for its good biocompatibility [7]. Therefore, the nanoparticle which contained SPIO and Cy5.5 may be used as a MR/fluorescence imaging contrast agent.

The optimization of both magnetic and fluorescence properties within a single probe is the key point because only improvement of either property seems to impair the other. In order to balance the fluorescence property and magnetic property in the probe, the certain interval between the emitting species and the magnetic material is of great importance [8]. Due to the short interval between the emitting species and the magnetic one, the fluorescence of the fluorophore would be strongly reduced or even quenched [9,10]. Therefore, different measures were taken to produce an interval between the fluorophore and magnetic one to obtain MR/fluorescence dual imaging probe. He et al. constructed a nanoprobe of silica-coated iron oxide, on which the surface was modified with fluorescence molecules [11]. In the probe, the silica coating can protect the fluorescence from SPIO's interference. Chen et al. reported that PLL-capped SPIO-PAA modified with NIR797 was used for MRI/fluorescence imaging [12]. PLL produced the interval between the SPIO and fluorescence molecule NIR797 in the nanoparticle. Therefore, it needs to control the interval between the emitting species and the magnetic materials to obtain a MR/fluorescence imaging probe.

Among a plurality of nanoparticulate systems, the polymeric micelle that formed through self-assembly of amphiphilic block copolymers has a core-shell structure. Its hydrophobic core serves as a natural carrier environment for hydrophobic drugs and the hydrophilic shell allows the particles' stability in an aqueous solution [13]. The stabilization of the polymeric micelles increases the circulation time *in vivo* and favors the preferential accumulation in tumors because of the enhanced permeability and retention (EPR) effect [14]. Moreover, the integration of PEG into the polymer micelles can reduce protein adsorption and limit immune recognition so as to increase the serum half-life of the nanoprobe *in vivo*[15].

Targeted delivery is an attractive strategy to further improve nanoparticle accumulation in tumor tissues and reduce side effects [16]. Transferrin (Tf) is one of the most widely used tumor-targeted ligands because Tf receptors (TfRs) are over-expressed on malignant cells and play a key role in cellular iron uptake through the interaction with Tf [17]. Moreover, the expression level of TfRs has been suggested to correlate with tumor stage or cancer progression [18-20].

To guide tumor excision, a tumor-targeted nanoprobe with the potential for pre-operation evaluation and intra-operation navigation is required to substantially improve the accuracy of surgical resection. Here we developed a water-dispersible hybrid nanoprobe wherein hydrophobic SPIONs were loaded into polymeric micelles through self-assembly of an amphiphilic block copolymer poly(ethylene glycol)-poly(ϵ-caprolactone) (PEG-*b*-PCL). Tf and Cy5.5 were conjugated onto the surface of the micelles to construct a targeted bi-functional probe SPIO@PEG-*b*-PCL-Tf/Cy5.5 (SPPTC). Then, the potential of the nanoprobe in MR and fluorescence imaging of tumors both *in vitro* and *in vivo* was evaluated.

## Methods

### Materials

All materials were purchased from commercial suppliers and used without further purification, unless otherwise noted. Cy5.5-NHS was purchased from GE Healthcare (Piscataway, NJ, USA). Succinimidyl iodoacetate (SIA), 2-iminothiolane hydrochloride (Traut's reagent), albumin from bovine serum (BSA), and Tf were purchased from Sigma-Aldrich (St. Louis, MO, USA). Methoxy-PEG_5000_-*b*-PCL_15000_ was purchased from Daigang Biomaterial Co., Ltd. (Jinan, Shandong, China). NH_2_-PEG_5800_-*b*-PCL_19000_ was purchased from Polymer Source Inc. (Montreal, Quebec, Canada). Other chemicals and reagents used in the study were of analytical grade.

All cell lines were preserved in our lab. BALB/c nude mice (male, 4 to 6 weeks) were purchased from Hunan Slake Jingda Experimental Animal Co. Ltd., Changsha, China (Animal Qualification Certification No. 43004700000595). All animal studies were approved by the Animal Experimentation Ethics Committee of College of Life Science and Technology, Huazhong University of Science and Technology and carried out in compliance with the guidelines approved by the Science and Technology Department of Hubei Province.

### Preparation of SPPTC

SPIONs were synthesized through the thermal decomposition method described previously [21]. SPIO-loaded polymeric micelles were prepared through a solvent evaporation method. Briefly, 6 mg of PEG-*b*-PCL (a mixture of mPEG-*b*-PCL and NH_2_-PEG-*b*-PCL (5:1)) and SPIONs (18 mg) were dissolved in THF (3 mL). Then the obtained solution was added slowly into 20 mL of distilled water in a dropwise manner under vigorous ultrasonic agitation. After, the beaker was exposed to air overnight, allowing evaporation of THF and formation of micelles slowly so as to obtain SPIO@PEG-*b*-PCL. A concentration of Cy5.5-NHS was adjusted to 20 mg/mL in DMSO. Cy5.5-NHS solution of 1.5 μL was added to 20 mL of the prepared SPIO@PEG-*b*-PCL solution, shaken for 2 h at room temperature. Then SIA (0.08 mg) in 40 μL DMSO was added to the Cy5.5 conjugated micelles and kept in the dark at room temperature for 2 h. The product was washed three times with and dissolved in distilled water (solution 1). Amount of 70 μL of a Traut's reagent (2 mg/mL) in sodium borate (0.15 M, pH 8.5) was added into 200 μL of 10 mg/mL Tf solution (20 mg, dissolved in 200 μL of 0.15 M sodium borate, pH 8.5) and kept at 4°C for 2 h (solution 2). Last, solution 1 was mixed with solution 2 and reacted at 4°C overnight. The final product was separated using a magnet by removing the supernatant.

### Characterization of SPPTC

The size and the morphology of SPPTC were characterized through transmission electron microscopy (TEM, JEM-2010, JEOL, Tokyo, Japan) at 200 kV. One drop of the sample solution (0.1 mg/mL) was deposited on a carbon-coated copper grid (200 meshes) and allowed to air dry. The excess solution was removed with filter paper. The hydrodynamic diameter and the size distribution of SPPTC were measured through dynamic light scattering (DLS, Zetasizer Nano ZS90, Malvern Instruments Ltd., Worcestershire, UK) at room temperature.

Magnetic properties were studied using vibrating sample magnetometer (VSM, ADE Model 4 HF VSM, ADE, Lowell, MA, USA) under the field of up to 15 kOe at room temperature. To determine the relaxivity, the nanoprobe was diluted in distilled water at an iron concentration range of 0 to 25 μg/mL. The samples were transferred to a 96-well plate, and T2 relaxation time was determined using a whole-body MR scanner (Signa HDx 3.0 T, GE, New York, NY, USA). The fluorescence properties were determined with IVIS® Lumina XR Imaging System (Caliper Life Sciences, Hopkinton, MA, USA).

### *In vitro* study

HL7702 cell line (normal human liver cell line) and HepG2 cell line (human liver tumor cell line) were cultured in DMEM (Gibco, Grand Island, NY, USA) and supplemented with 10% fetal bovine serum (Gibco, Grand Island, NY, USA), penicillin (100 mg/mL), and streptomycin (100 mg/mL) in a humidified atmosphere with 5% CO_2_ at 37°C.

### Reverse transcription-polymerase chain reaction analysis of TfR1

The PCR primers of TfR1 and GAPDH were designed according to the literature [22]. The TfR1 expression level was determined in HepG2 cell and HL7702 cell. The mRNA of GAPDH was used as the control in each test cell line. PCR consisted of initial denaturation at 94°C for 3 min; 30 three-step cycles at 94°C for 1 min, 53°C for 1 min, and 72°C for 1 min; and a final extension at 72°C for 10 min.

### *In vitro* MRI imaging

HL7702 cells and HepG2 cells were incubated with SPPTC at different iron concentrations (0, 5, 10, 15, 20, and 25 μg/mL) at 37°C for 2 h. After, the cells were washed with PBS (0.1 M, pH 7.4) three times and dispersed in PBS, and then the cells were dispersed with 350 μL of 1% agarose gel per sample for MRI with 3.0-T whole-body MR scanner.

### *In vitro* fluorescence imaging

The cellular uptake of SPPTC was determined using confocal microscope. The HepG2 cells were inoculated in 6-well plates at a density of 1 × 10^5^ cells per well and cultured at 37°C overnight. After washing with PBS, the cells were incubated with SPIO@PEG-*b*-PCL-Cy5.5 (SPPC) and SPPTC (Fe, 20 μg/mL), respectively, for 2 h in a CO_2_ incubator. After incubation, the cells were washed with PBS three times to remove the remaining nanoprobes and fixed in a 4% formaldehyde/PBS solution for 20 min. Then the fixative was removed with PBS. The fluorescence imaging results were obtained through a Leica laser scanning confocal microscope (Leica TCS-SP5, Leica, Wetzlar, Germany).

### *In vivo* studies

#### Induction of subcutaneous HepG2 tumor

Male BALB/c nude mice bearing subcutaneous HepG2 tumor xenografts were used as models. The subcutaneous xenografts were generated by subcutaneously injecting 6 × 10^6^ HepG2 cells into the back leg of the nude BALB/c mouse. The mice were used for the following imaging analyses when the tumors reached a diameter of approximately 1 cm.

### *In vivo* fluorescence imaging

The nude mice bearing the tumor were assessed for Cy5.5 emission at a wavelength of 640 nm after intravenous injection of SPPC or SPPTC suspension (10 mg/kg Fe of body weight). During fluorescence imaging, mice (*n* =6) were anesthetized using gas with a mixture of oxygen and isoflurane (Hebei Jiupai Pharmaceuticals, Shijiazhuang, Hebei, China). The images were taken at different time points (0, 4, and 8 h) after intravenous injection of SPPC or SPPTC. The images were captured using IVIS® Lumina XR Imaging System.

### *In vivo* MRI

The animals were scanned under Intera 3.0 T MR scanner (Philips, Amsterdam, Netherlands) after fluorescence imaging. The T2-weighted echo images were acquired before and after nanoprobes' administration. The following parameters were used: field of view (FOV) =120 mm; base resolution =192 × 160; repetition time (TR) =2,000 ms; echo time (TE) =20, 40, 60, 80, 100, 120, and 140 ms; slice thickness =1.5 mm. The MRI T2 signal intensities within the region of interest (ROI) and noise signal were measured before and after administration of the probe. The change of T2 signal value was calculated according to the following formula:

$$\begin{array}{l} \left[\left(SI_{\mathrm{post},\mathrm{tumor}}/SI_{\mathrm{post},\mathrm{noise}}\right)-\left(SI_{pre,\mathrm{tumor}}/SI_{pre,\mathrm{noise}}\right)\right]/\\ \;\left(SI_{pre,\mathrm{tumor}}/SI_{pre,\mathrm{noise}}\right)\times 100\% \end{array}$$

After MR imaging, each group of the mice was sacrificed and the tumor tissues were further processed for Prussian blue staining.

### Prussian blue staining

The tumor tissues were fixed in 10% formalin and cryoprotected with 18% sucrose solution. The sections were incubated for 30 min in a solution of 2% potassium ferrocyanide and 2% hydrochloric acid at a 1:1 ratio. After Prussian blue staining, the sections were counterstained with 1% nuclear fast red solution.

### Cell toxicity

Normal human liver HL7702 cells (HL7702) were used for cytotoxicity study. Briefly, the HL7702 cells were cultured in 96-well plates and kept overnight for cell attachment. After then, a medium containing SPPTC was added in a dilution series (medium containing 0, 15, 30, 45, and 60 μg/mL Fe). The culture medium was added into the control well. After 24, 48 and 72 h of incubating the cells with the materials, 20 μL of 3-(4,5-dimethylthiazol-2-yl)-2,5-diphenyltetrazolium bromide (MTT) (5 mg/mL) was added to each well. After incubation for 4 h, the formazan crystals were solubilized with 150 μL of DMSO. The absorbance of each well was measured using a microplate reader (1420 multilabel counter, PerkinElmer, Waltham, MA, USA) at 490 nm to determine the relative cell viability.

### Statistical analysis

Statistical analysis was performed using student's *t*-test using statistical software (SPSS 13.0, Chicago, IL, USA). A difference of *p* <0.05 was considered statistically significant.

## Results and discussion

### Construction and characterization of nanoprobe

**Figure 1:**
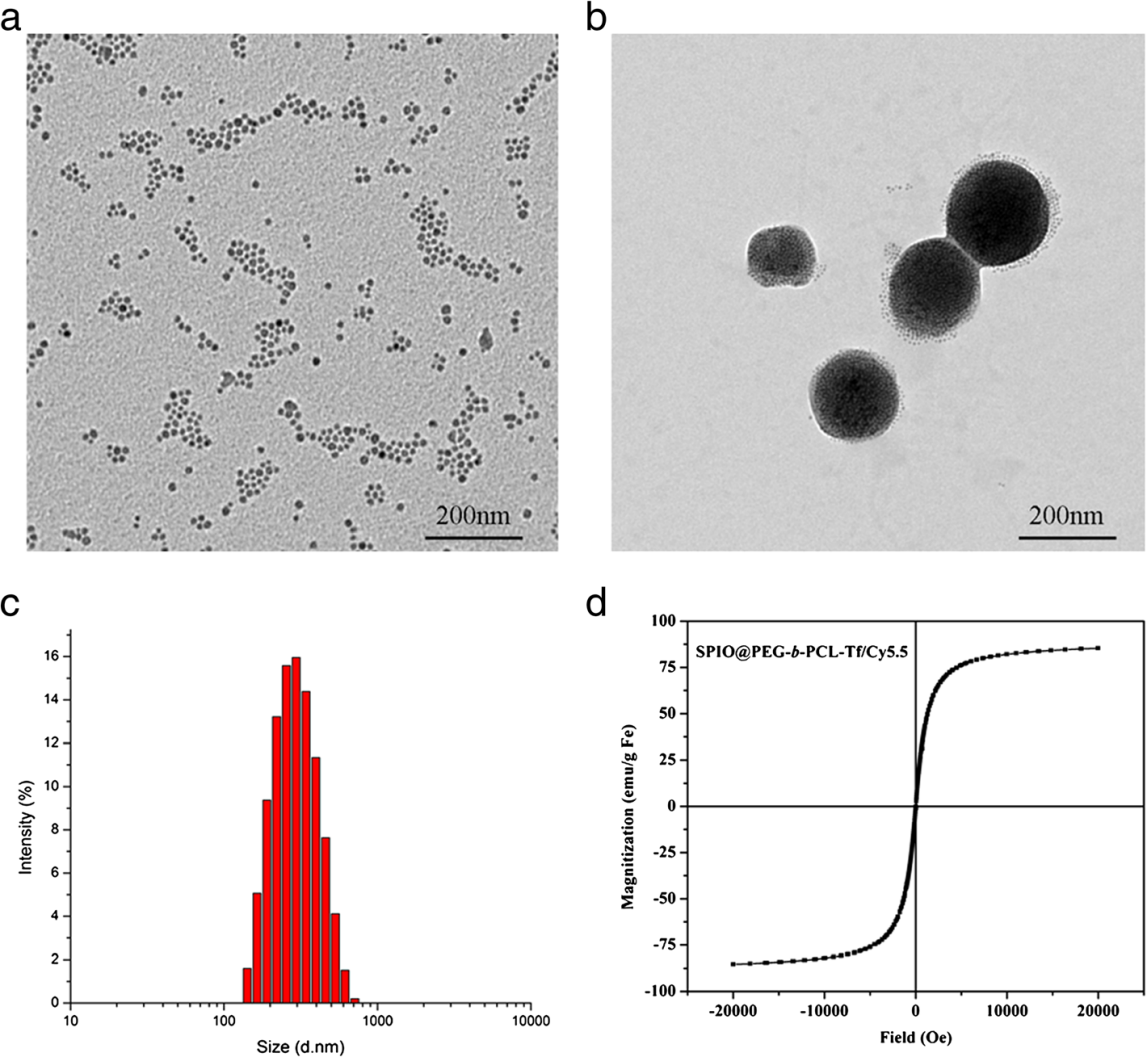
**Characterization of SPPTC.** TEM images of SPIO **(a)**; TEM images of SPPTC through negative staining **(b)**; hydrodynamic size distribution of SPPTC about 196 nm with PDI 0.168 **(c)**. The saturation magnetizations (Ms) of SPPTC was 85.4 emu/g Fe at 300 K **(d)**.

From the transmission electron microscope images, the average size of Fe_3_O_4_ nanoparticles (SPIONs) prepared through thermal decomposition method was about 10.2 nm (Figure 1a). After the SPIONs were being encapsulated into the micelles, the size of SPIO@PEG-*b*-PCL was approximately 169.3 nm (Figure 1b). The results of DLS showed that the hydrodynamic diameter of the SPIO@PEG-*b*-PCL was about 196 nm with PDI of 0.168 (data not shown). The hydrodynamic diameter of the micelles increased (approximately 274 nm) after being conjugated with Cy5.5 and Tf (Figure 1c).

### Magnetic and fluorescence properties of SPPTC

**Figure 2:**
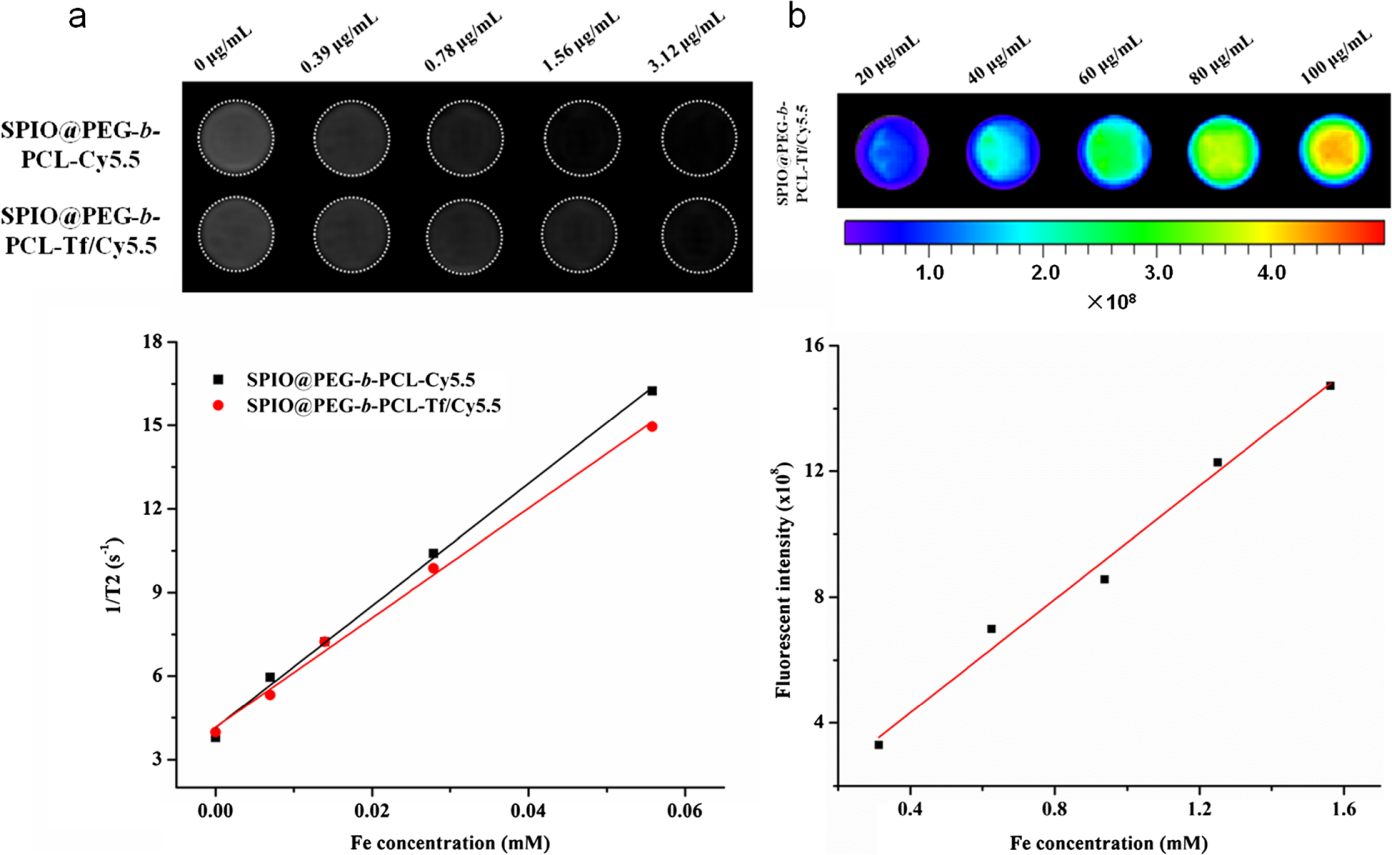
**Magnetic and fluorescent properties of SPPTC.** The r2 of SPPTC is 196.5 mM^−1^ s^−1^ and the r2 of SPPC is 219.1 mM^−1^ s^−1^ at 300 K respectively **(a)**; the correlation between fluorescence intensity and the concentration of the nanoprobe **(b)**.

Magnetic properties were important parameters for an MRI contrast agent. The magnetization of SPPTC was measured at room temperature. At 300 K, SPPTC exhibited superparamagnetic behaviors, with zero coercivity and remanence. The saturation magnetization of the material was 85.4 emu/g (Figure 1d). In addition, the MR imaging capabilities of SPPTC and SPPC were evaluated at 3.0 T through acquiring phantom MR images of the agarose sample. The signal intensity of SPPTC and SPPC decayed significantly with the increasing iron concentration (Figure 2a). The relaxivities of SPPTC and SPPC were 196.5 and 219.1 mM^−1^ s^−1^ respectively, both showing a favorable contrast effect. These high relaxivities may be due to the cluster of SPIO formed in the micelles. Ai et al. had reported that a cluster of SPIO particles had much higher T2 relaxivity compared the T2 relaxivity of single SPIO [23]. The fluorescence intensity increased along with the increase of the nanoprobe concentration (Figure 2b). It demonstrated that the fluorescence was protected from the bleaching of SPIO because of the interval produced by PEG-*b*-PCL. The results indicated that the magnetic/fluorescence micelles could function as an efficient imaging probe.

### TfR1 expression analysis

The evaluation of the TfR1 expression level in HL7702 and HepG2 cells was carried out by reverse transcription-polymerase chain reaction (RT-PCR). The results showed that the expression of TfR1 mRNA in the HepG2 cell line was obvious but there was almost no expression in the normal cell line HL7702 (Additional file 1: Figure S1). The TfR family includes TfR1 and TfR2. The affinity of TfR1 for Tf is approximately 30-fold higher than the affinity of TfR2 [18]. Most cells can effectively control iron uptake by regulating TfR1 expression [17]. Iron overload conditions contribute to several pathological conditions including iron overload-related hepatic cancer [24]. The TfR expression level is often higher in the tumor tissue than the normal tissue [20]. Therefore, Tf can be used as the ligand for targeting liver tumor.

### *In vitro* studies

**Figure 3:**
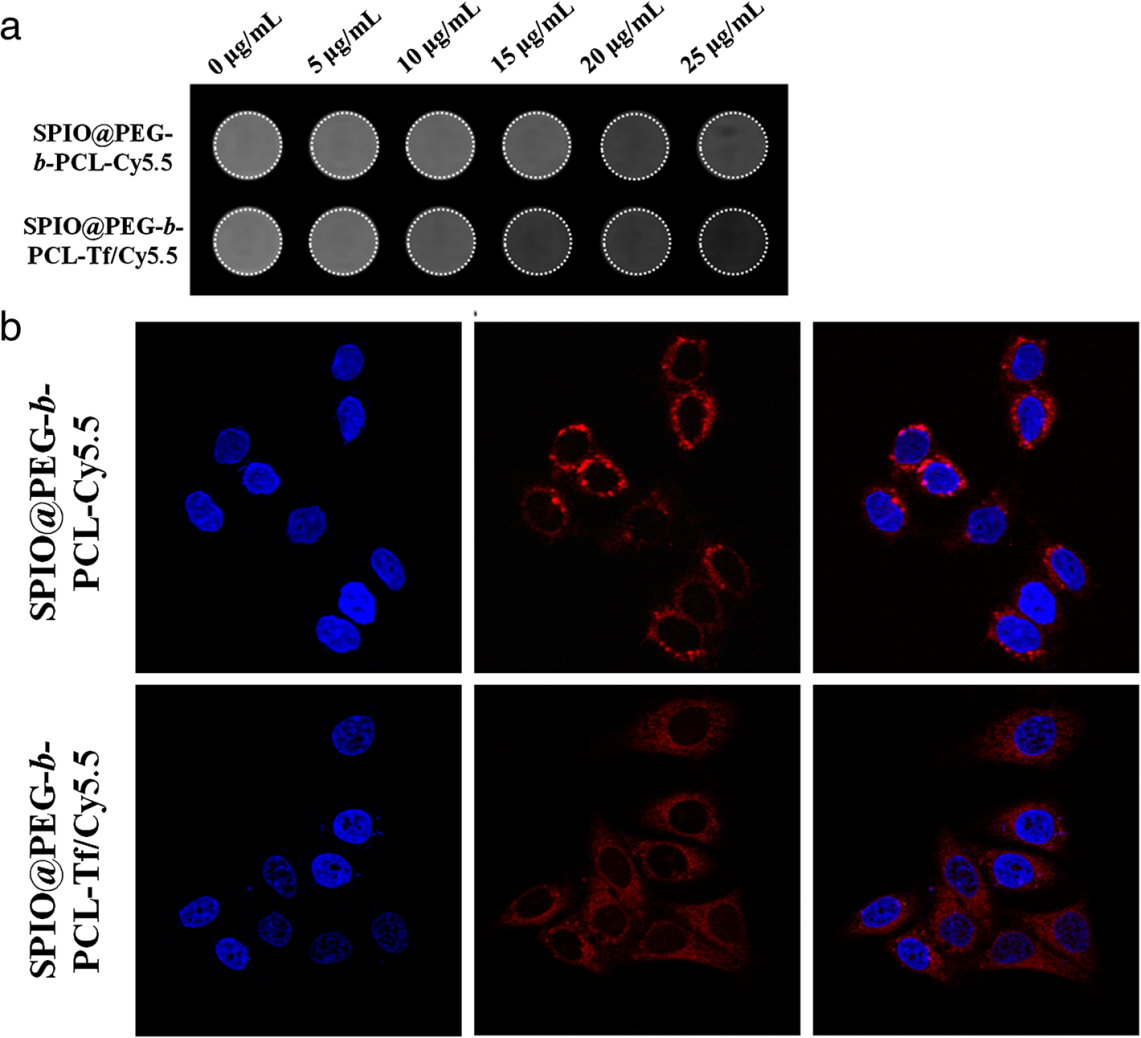
**The uptake of SPPC or SPPTC in HepG2 cell at different concentrations (Fe).** Detected through MRI **(a)**; detected by confocal microscope **(b)**.

From the MR imaging, significant gradual MR signal decays in HepG2 cells along with the increasing iron concentration when the HepG2 cells were treated with SPPTC or SPPC. However, the signal in the cells treated with SPPTC decayed much greater than that of the cells treated with SPPC at the same iron concentration (Figure 3a). The strong fluorescence signal could be observed throughout the cytoplasm in HepG2 cells when treated with SPPTC, while a weak fluorescence signal existed in the HepG2 cells treated with SPPC (Figure 3b). Therefore, SPPTC can be used as MR/fluorescence imaging contrast agent in HepG2 cell.

The nanoprobes with magnetic and fluorescence properties are of special interest in monitoring and manipulating at the same time. Chen et al. reported a nanoprobe in which NIR979 conjugated onto the surface of PLL-capped SPIO-PAA nanoparticle was used for dendritic cell tracking [12]. Wen et al. reported cationic polymersomes loaded with SPIONs and QDs were used to label neural stem cells [25]. Gollavelli et al. reported dual model magnetic and fluorescence imaging of fluorescent grapheme (MFG)-SiNc4 in Hela cells [26]. Here SPPTC can be used to detect liver tumor cell.

### *In vivo* studies

**Figure 4:**
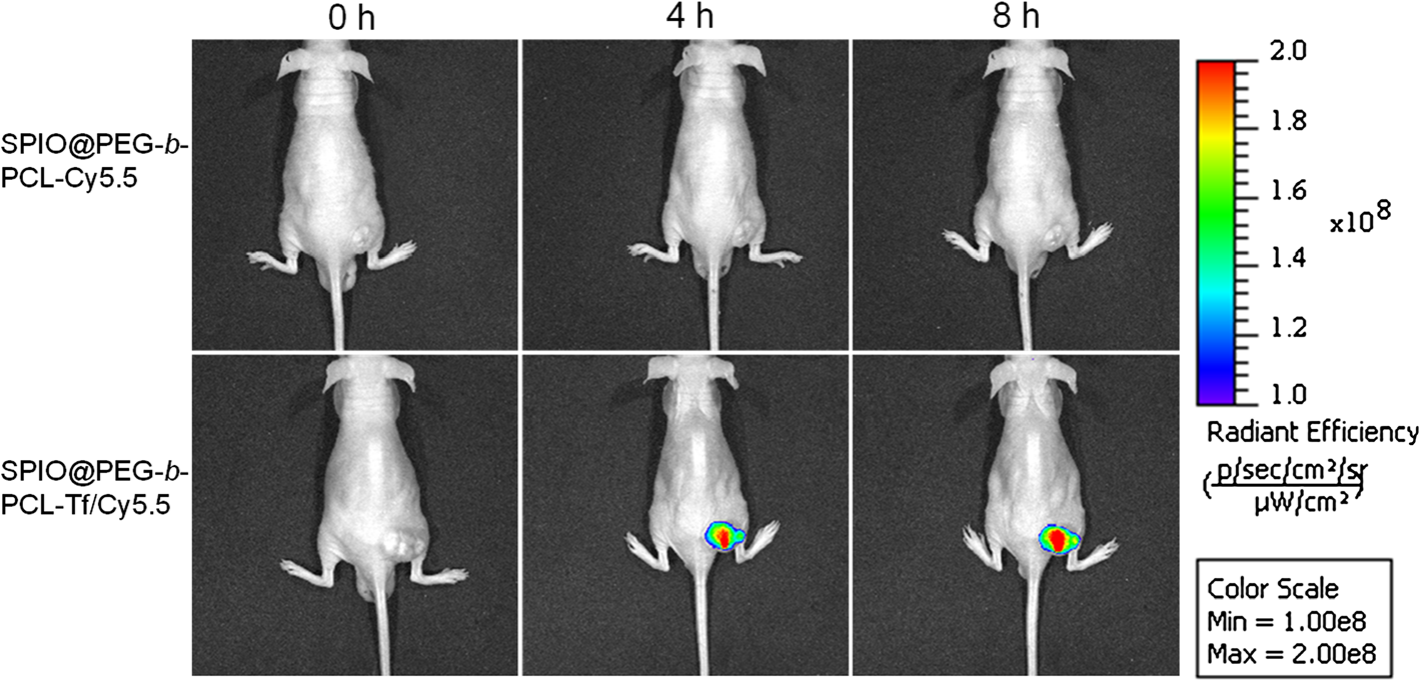
**Fluorescence imaging of SPPTC in mice bearing the subcutaneous tumor at different time points.** Top row, injected with SPPC; bottom row, injected with SPPTC.

**Figure 5:**
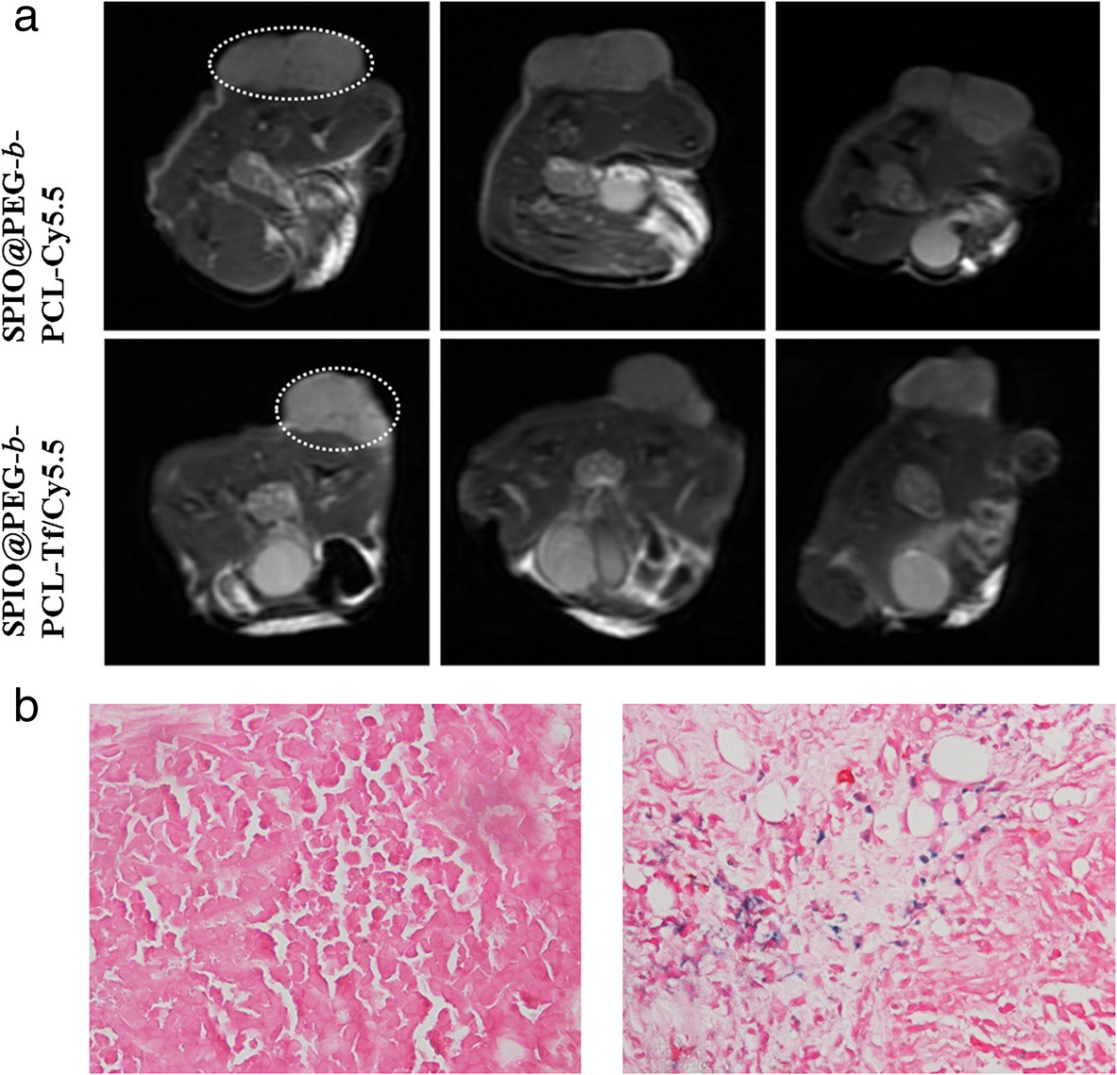
**T2-weighted MR imaging and Prussian blue staining.***In vivo* T2-weighted MR imaging before and after intravenous injection of SPPC or SPPTC **(a)**; the histological sections of tumor with Prussian blue staining **(b)**.

The *in vivo* fluorescence images at the pre- and post-injection were shown in Figure 4. At different time points after intravenous injection of SPPTC, strong fluorescence signals could be observed at the tumor site, while no significant fluorescence could be detected at the tumor site after injection with SPPC. Besides the site of tumor, the fluorescence signal can be detected at the region of liver or spleen after being taken out of the mice (Additional file 2: Figure S2), which may be due to the unspecific uptake of the reticuloendothelial system (liver or spleen). The ability of SPPTC as a MRI contrast agent was further assessed in the mouse model. The MR images of the tumor site displayed significant contrast enhancement after treated with SPPTC (Figure 5a). The MR signal intensity at the tumor site decayed up to 54% in SPPTC-treated mice compared to the pre-injection MR images, while it was 16% in the SPPC group (data not shown). To further verify the accumulation of SPPTC at the tumor site, tumor tissue slices were stained with Prussian blue for ferric ions and by a nuclear fast red solution for the cell nucleus. As shown in Figure 5b, the accumulation of SPIONs could be clearly observed in the tumor tissues. In contrast, no apparent accumulation was detected in the SPPC-treated group.

Surgery remains the gold standard for clinical treatments of most tumors, but resection bears the potential risk of incomplete excision due to the invasiveness of the malignant tumor. The most important potential application of the dual MR/fluorescence imaging nanoprobe is to provide help in pre-operation evaluation and intra-operation navigation in clinic. There are a variety of approaches for the synthesis of nanoparticles with fluorescent and magnetic properties. Ni et al. reported an upconversion nanoprobe (ANG/PEG-UCNPs) for MR/fluorescence imaging of glioblastoma in mice [27]. Zhang et al. reported a dual functional nanoprobe to which anti-EGFR mAb and Cy5.5 were conjugated onto PEG-coated Fe_3_O_4_ nanocrystals was used to detect the mouse mammary tumor [28]. In our previous work, we had constructed the pH-/temperature-sensitive magnetic nanogels conjugated with Cy5.5-labeled lactoferrin for MR and fluorescence imaging of glioma in rats [29]. Veiseh et al. reported an iron oxide nanoparticle coated with a PEGylated chitosan-branched copolymer to which chlorotoxin and Cy5.5 were conjugated for brain tumor imaging [15]. As far as we know, there are few reports about SPIO coated with micelles, conjugated with Tf and Cy5.5 on the surface and provided for dual model imaging in liver tumor.

### Cell toxicity studies

**Figure 6:**
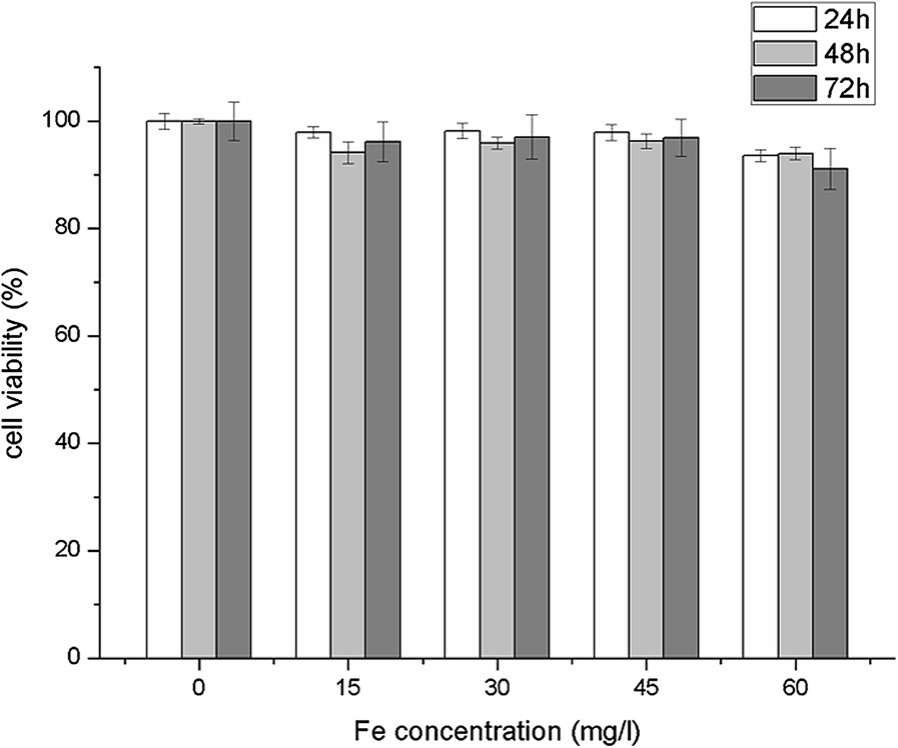
Viabilities of HL7702 cells treated with SPPTC.

The excellent biocompatibility of SPPTC was verified *in vitro*. From Figure 6, no significant change of activity in the normal liver cell line (HL7702 cell line) was found when the cells were incubated with SPPTC at a concentration range of 0 to 60 μg/mL (Fe concentration).

## Conclusions

In the present study, the magnetic/fluorescence polymeric micelles are easily prepared and well-tolerated and remain favorable MRI and fluorescence imaging ability both *in vitro* and *in vivo*. Therefore, SPPTC had the potential application in pre-operation evaluation by MR imaging and guiding of tumor resection by intra-operative fluorescence imaging.

## Abbreviations

DMEM: Dulbecco's modified Eagle's medium; MRI: magnetic resonance imaging; PEG-*b*-PCL: poly (ethylene glycol)-poly (ϵ-caprolactone); PCR: polymerase chain reaction; SPIONs: superparamagnetic iron oxide nanoparticles; SPPTC: SPIO@PEG-*b*-PCL-Tf/Cy5.5; SPPC: SPIO@PEG-*b*-PCL-Cy5.5; Tf: transferrin.

## Competing interests

The authors declare that they have no competing interests.

## Authors’ contributions

HQ, ZL, and KD carried out the experiments. KM, SL, and QZ participated in the animal experiment. YZ conceived the study. YZ, WZ, and XY designed the experiments. YZ, HQ, and ZL participated in the modification of the article. All authors read and approved the final manuscript.

## Supplementary Material

Additional file 1: Figure S1 Expression of TfR1 in HepG2 cell not in normal liver cell HL7702. GAPDH was used as the control. Click here for file

Additional file 2: Figure S2 Fluorescence images of different organs after injection of SPIO@PEG-*b*-PCL-Tf/Cy5.5 at 24 h. Click here for file
