# Supplementary figures and images for: Transferrin-targeted magnetic/fluorescence micelles as a specific bi-functional nanoprobe for imaging liver tumor
Source: Nanoscale Res Lett. 2014 Oct 30;9(1):595. doi: 10.1186/1556-276X-9-595 (PMC4228372; doi:10.1186/1556-276X-9-595)

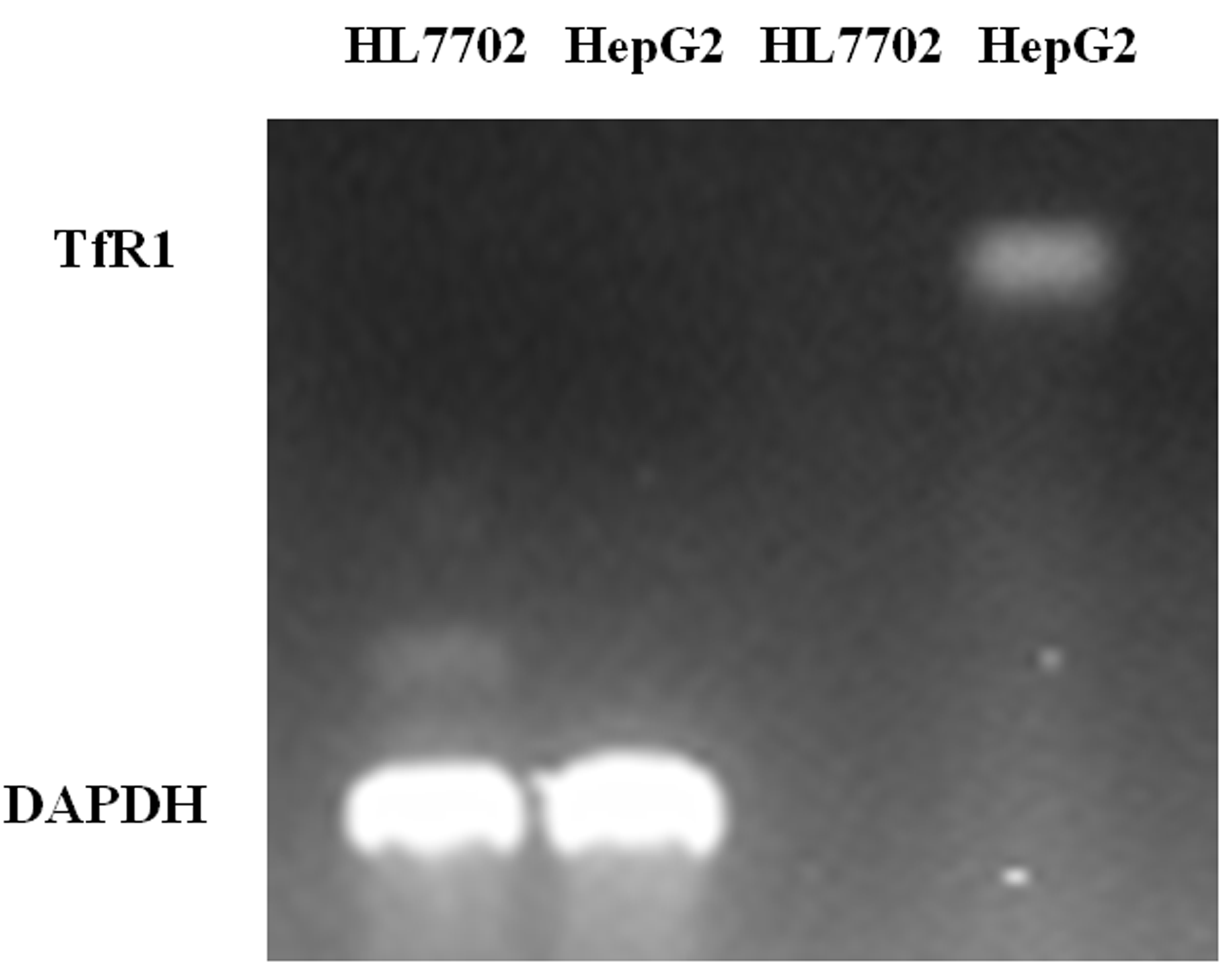

Supplement: Additional file 1: Figure S1 — Expression of TfR1 in HepG2 cell not in normal liver cell HL7702. GAPDH was used as the control. [file 1556-276X-9-595-S1.tiff]

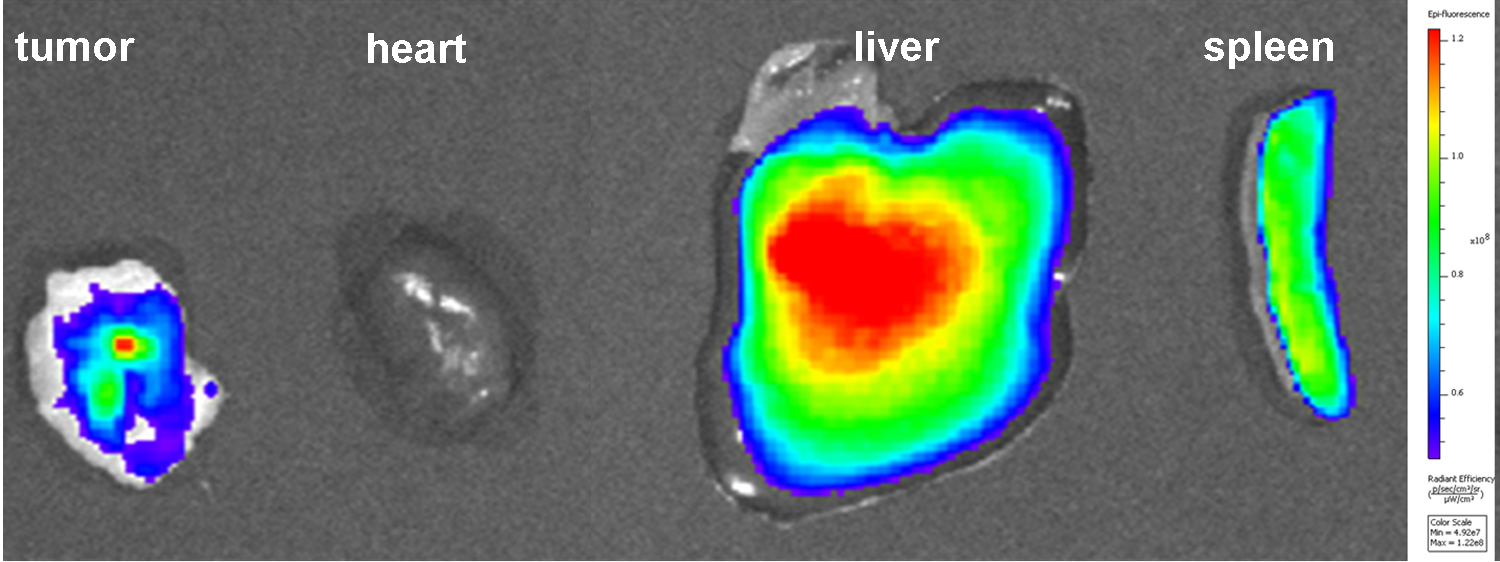

Supplement: Additional file 2: Figure S2 — Fluorescence images of different organs after injection of SPIO@PEG-b-PCL-Tf/Cy5.5 at 24 h. [file 1556-276X-9-595-S2.tiff]
